# Similar femoral stem fixation but less metaphyseal loss of bone mineral density with a taper-wedge design and diaphyseal bone preservation with a long and round-tapered design: a 5-year randomized RSA and DXA study of 50 patients

**DOI:** 10.2340/17453674.2025.43907

**Published:** 2025-09-02

**Authors:** Peter Bo JØRGENSEN, Morten HOMILIUS, Daan KOPPENS, Torben Bæk HANSEN, Maiken STILLING

**Affiliations:** 1 Department of Orthopaedics, University Clinic for Hand, Hip and Knee Surgery, Gødstrup Hospital; 2 Department of Orthopaedics, Aarhus University Hospital; 3 Department of Clinical Medicine, Aarhus University, Denmark

## Abstract

**Background and purpose:**

The new Tri-Lock bone -preserving stem with a collarless proximal-coated tapered-wedge design was compared with a classic well-proven collarless proximal-coated long and round-tapered design. Our primary aim was to compare femoral stem fixation (subsidence) of the Tri-Lock stem with the classic Summit stem, and secondarily to compare the change in periprosthetic bone mineral density (BMD) and PROMS between stem groups.

**Methods:**

In a patient-blinded randomized controlled trial, 52 patients at mean age 60 (SD 6) received cementless Tri-Lock (n = 26) or Summit (n = 26) femoral stems with a cementless Pinnacle cup, a cross-linked polyethylene liner, and a CoCr head. Patients were followed for 5 years with radiostereometric analysis (RSA), dual-energy X-ray absorptiometry (DXA), and patient-reported outcome measures (PROMs). We measured mean (CI) values of migration and periprosthetic bone mineral density and calculated between group differences.

**Results:**

At 2-year follow-up, the mean difference in subsidence was 0.14 mm (95% confidence interval [CI] –0.27 to 0.56) and below the chosen minimal clinically important difference of 0.6 mm. At 5-year follow-up, for the Tri-Lock and Summit stems, the mean subsidence was 0.38 (CI 0.04–0.72) and 0.24 (CI 0.09–0.57), and the mean retroversion was 1.68° (CI 0.80–2.55) and 1.53° (CI 0.68–2.37), respectively. There was initial periprosthetic BMD loss for both stems. At 5-year follow-up, the mean metaphyseal bone loss was minimal for the Tri-Lock stem (zone 1: –2.8% vs –11.5%) while the Summit stem preserved the medial diaphyseal bone better (zone 6: –7.1% vs –13.6%). At the medial stem tip, BMD was increased with the Summit stem (zone 5: +3.4% vs –1.5%). At 5-year follow-up, median EQ5D was 1 in both groups and median Oxford Hip Score was 47 (Tri-Lock) and 45 (Summit) with no statistical significant differences between groups.

**Conclusion:**

The Tri-Lock and the Summit stems displayed similar migration until mid-term follow-up. At 3 months both stems had lost metaphyseal periprosthetic bone mineral density (BMD). During the following years, the new design regained more metaphyseal BMD. Contrarily, the long and round-tapered stem design regained or even increased diaphyseal BMD. PROM scores improved beyond the reference level for both groups.

In total hip arthroplasty the main challenges are early complications and in the longer term aseptic loosening related to bone loss. Bone loss may be influenced by the stem design. Tapered-wedge stem design is intended for proximal fixation in the metaphyseal area, whereas a classic round-tapered and longer stem may gain fixation in the diaphyseal region. Longer and round-tapered stems may pose a risk of hoop stress on the bone and a risk of femoral bone fracture [1]. However, some studies have shown an increased risk of periprosthetic fracture in shorter stems with proximal fixation [2]. Tapered-wedge stem designs have a shorter and curved stem with a reduced lateral shoulder intended for bone preservation.

Several factors may affect the periprosthetic bone and the fixation of cementless femoral stems after surgery. Ultra-porous coatings applied to the metaphyseal stem region increase the surface area for osseointegration [3]. Titanium alloy has an elastic modulus close to that of bone and has been associated with low periprosthetic bone resorption [4]. However, as the elasticity of titanium femoral stems also relates closely to stem size the issue is complex [5]. Furthermore, the bone microstructure and density of the patient’s femur are essential to support implant fixation, and cementless femoral stems have been shown to migrate more in patients with osteopenia and osteoporosis [6].

Factors for the long-term risk of stem loosening and subsequent revision are stem migration and bone loss. Migration can be evaluated using radiostereometric analysis (RSA) as a validated surrogate marker. Early migration of cementless stems is expected, but the amount of short-term stem migration and the migration pattern over time may predict stem survival [7-9]. For femoral stems, undesirable migration patterns of clinical importance include stem subsidence and stem rotation (version) [7,10]. From the 2-year follow-up, cementless stems should be stable [11]. Bone loss can be measured using dual-energy X-ray absorptiometry (DXA). Periprosthetic bone loss due to stress shielding is closely related to implant design and fixation, and may also be a predictor for hip implant loosening [12].

A phased introduction of new implants, including safety studies of implant fixation compared with a gold standard, has been proposed [13]. The Summit stem has a 10-year survival of 97.5% in national registries and can be considered a well-proven standard for a classic longer and round-tapered cementless stem [14]. The Tri-Lock stem was marketed in 2008. The short-term clinical results of the Tri-Lock stem were promising, but implant fixation was insufficiently explored.

Our primary aim was to compare femoral stem fixation (subsidence) of the newer metaphyseal ultra-porous-coated taper-wedge Tri-Lock stem with the classic metaphyseal porous-coated longer round-tapered Summit stem, and secondarily to compare the change in periprosthetic bone mineral density (BMD) and PROMS between stem groups.

## Methods

### Study design

This study was a patient-blinded randomized study with evidence level II.

All patients gave written consent to study participation at Gødstrup Hospital between January 2015 and August 2017. Inclusion criteria were: (i) primary hip osteoarthritis, (ii) sufficient bone quality for cementless implants, assessed by the surgeon, and (iii) age between 40 and 70 years. Exclusion criteria were: (i) neuromuscular or vascular diseases in the affected leg, (ii) patients who could not refrain from the use of non-steroidal anti-inflammatory drugs (NSAIDs) in postoperative recovery, (iii) preoperative hip fracture sequelae, (iv) women planning pregnancy during the study period, and (v) hip dysplasia or sequelae to Legg–Calve–Perthes disease. Patients were block randomized in blocks of 10 to the Tri-Lock (n = 26) or the Summit (n = 26) femoral stem group. In the theater, just before surgery, the randomization was revealed by drawing consecutively numbered concealed opaque envelopes. Data was analyzed using an intention-to-treat approach and the study is reported according to CONSORT guidelines.

### Follow-up schedule

Baseline stereoradiographs and periprosthetic BMD were obtained postoperatively. Baseline patient-reported outcome measures and systemic BMD were recorded preoperatively. Stereoradiographs, periprosthetic BMD, and patient-reported outcome measures were further collected at 3-month, 1-, 2-, and 5-year follow-up.

### Sample size

A sample size calculation aimed to detect a minimal clinically relevant difference of 0.6 mm (SD 0.7) for stem subsidence at 2-year follow-up. A t-test with a power of 80% and an alpha value of 0.05 revealed a need for 23 patients in each group [6,15]. To account for technical errors in measurements and patient dropouts we planned the inclusion of 50 patients. In total, 52 patients were included to account for 2 early dropouts during the inclusion period.

### Implants

**Figure 1:**
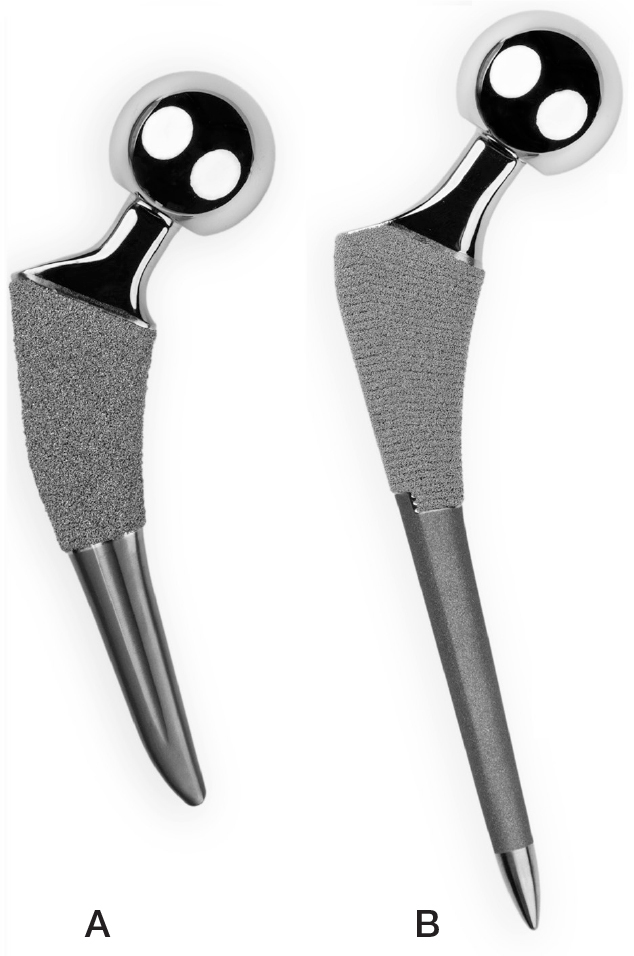
Tri-Lock (A) and Summit (B) femoral stems.

The Tri-Lock Bone Preservation Stem (Depuy Orthopedics, Inc, Warsaw, IN, USA) (Figure 1) is a short stem that was introduced in 2008. It is redesigned from the original Tri-Lock stem and, compared with the original Tri-Lock stem, the Tri-Lock Bone Preserving Stem is shorter and has a reduced lateral shoulder to preserve bone in the greater trochanter [16]. The Tri-Lock femoral stem is a cementless collarless tapered-wedge stem design with an oblique tip cut. The stem is intended for metaphyseal fixation supported by a proximal titanium coating with Gription (Depuy Orthopedics, Inc, Warsaw, IN, USA), with a volume porosity of 63% and a coefficient of friction of 1.2% [3].

The Summit femoral stem (Depuy Orthopedics, Inc, Warsaw, IN, USA) is a cementless collarless round-tapered classic length straight stem design. The distal part is grit-blasted with a polished tip [17].The stem is also intended for metaphyseal fixation supported by a proximal coating Porocoat (Depuy Orthopedics, Inc, Warsaw, IN, USA), which is made from titanium and has a volume porosity of 45% and coefficient of friction of 0.8 (Figure 1) [3]. The stem also has some diaphyseal support but is described to have a low risk of hoop stress and femoral fracture [18].

Both stems are made from titanium alloy.

All patients received a cementless porous coated titanium Pinnacle cup (Depuy Orthopedics, Inc, Warsaw, IN, USA), a cross-linked polyethylene liner (Marathon, Depuy Orthopedics, Inc, Warsaw, IN, USA), and a 32 mm or 36 mm CoCr femoral head (Depuy Orthopedics, Inc, Warsaw, IN, USA).

### Surgical approach and discharge

All patients were operated on by 1 of 3 experienced hip surgeons. Tranexamic acid (1 g) was administered for bleeding prophylaxis before operation. For infection prophylaxis, cefuroxime (1.5 g) was administered preoperatively and 3 times postoperatively with 8-hour interval. For thrombo-prophylactic treatment, patients received low-molecular-weight heparin until discharge. All patients were operated on using a posterolateral approach. For RSA, 7 tantalum markers (1 mm) were inserted in the femur (4 in the greater trochanter and 3 in the lesser trochanter). Preparation of the femoral canal for the Summit stem included reaming of the greater trochanter for correct centralization/insertion of the longer straight stem. For the Tri-Lock stem only broaching of the femoral canal with complete preservation of the greater trochanter was used. All patients followed the same fast-track discharge program, aiming for discharge within 24 hours after operation, with pre- and postoperative physiotherapy and 6-week follow-up.

### Radiostereometric analysis (RSA)

All patients were mobilized with full weightbearing on the first postoperative day. Postoperative stereoradiographs were taken within 1–2 days postoperatively, before discharge [19]. Digital stereo radiographs (RSA) were recorded with the patient supine using an RSA setup (NRT X-ray Technique, Aarhus, Denmark) [14].

Stem migration was measured as translation and rotation of the origin and coordinate system of the stem relative to bone markers in the femur (see Supplementary Figure 1). We used the same markers as reference for all follow-ups and data for left-side hips was normalized to a right-side coordinate system [20]. The postoperative stereoradiograph was used as reference in the migration analysis, which was performed with Model-Based RSA (RSAcore v. 4.2, Leiden, The Netherlands). We used a combined femoral stem model with a sphere for the femoral head and a CAD model of the stem. The origo of the model along with the coordinate system is presented in Supplementary Figure 1. The rigid body error threshold was 0.35 mm. The mean condition number (CN) was 63 (range 34–101) and the maximum accepted CN was 120 [20]. No stems were excluded due to a high condition number. At the 3-month follow-up, double examinations were acquired to calculate the RSA precision and limit of agreement (1.96*SD) (Supplementary Table 1).

### T-score and BMD changes

DXA scans for measurement of bone mineral density (BMD) were performed using a GE Lunar iDXA scanner (General Electric, Chicago, IL, USA), and analyzed using encore software (v. 13, General Electric, Chicago, IL, USA). Preoperative systemic BMD was assessed in L1–L4 and total hip and given as a T-score for the lowest value obtained. Patients with a T-score ≤ –2.5 SD were referred to the department for osteoporosis. Postoperative DXA scans of the hip were obtained within 1–2 days after surgery and used as reference to assess periprosthetic percentual BMD changes in 7 Gruen zones around the stem [21]. At the 3-month follow-up, double examinations were acquired to calculate BMD precision (Supplementary Table 2).

### PROMS

EQ-5D was indexed using the Danish Time Trade-Off value set [22] with a minimal clinically important difference (MCID) of 0.3 [23]. Oxford hip score (OHS) was calculated ad modum Murray [24] and we used an MCID of 5 points [23]. Pain during activity and rest was reported by patients on a 0 to 10-point numeric scale. Complications in terms of revision (and reasons for revision) and dislocations were registered prospectively during the study.

### Statistics

Normal distribution of data was confirmed using probability plots. The hypothesis of no difference for both the primary outcome (group difference in stem subsidence at 2-year follow-up) and the secondary outcomes (group differences in periprosthetic BMD changes and improvement of patient-reported outcomes) was analyzed using univariate repeated measurement analysis (mixed model) with follow-up time and stem type as fixed effect allowing for interaction, and patient as random effect. Normality and homoscedasticity of the mixed model residuals were examined at both the measurement and the random effects levels, using Q–Q plots to assess normality and plots of fitted values versus residuals to inspect constant variance. Correlation between z-score standardized RSA and BMD measures was performed using least-square linear regression and residual distribution was evaluated using histogram and scatterplots. Standardized correlation coefficients (β) were presented with corresponding 95% confidence intervals (CI). Changes in Oxford Hip Score and EQ5D were compared between the 2 groups using pairwise group comparisons to describe differences (Student’s t-test, two-tailed). 2 patients were excluded from the dataset due to periprosthetic fracture (n = 1, Tri-Lock) and infection (n = 1, Tri-Lock).

Statistical significance was assumed at P < 0.05. Statistical calculations were performed using Stata (IC 17, StataCorp LLC, College Station, TX, USA).

### Ethics, registration, funding, data sharing, and disclosures

The study was conducted in accordance with the Helsinki II declaration, and all patients gave informed written consent prior to participation. Approvals were obtained from the local ethics committee [1-10-72-536-12] and registered at ClinicalTrials.gov [NCT06019026] and the Danish Data Protection Agency [1-16-02-42-24]. DePuy paid for the software license of the CAD models used in the RSA analysis but had no influence on the publication of the study. Complete disclosure of interest forms according to ICMJE are available on the article page, doi: 10.2340/17453674.2025.43907

## Results

**Figure 2:**
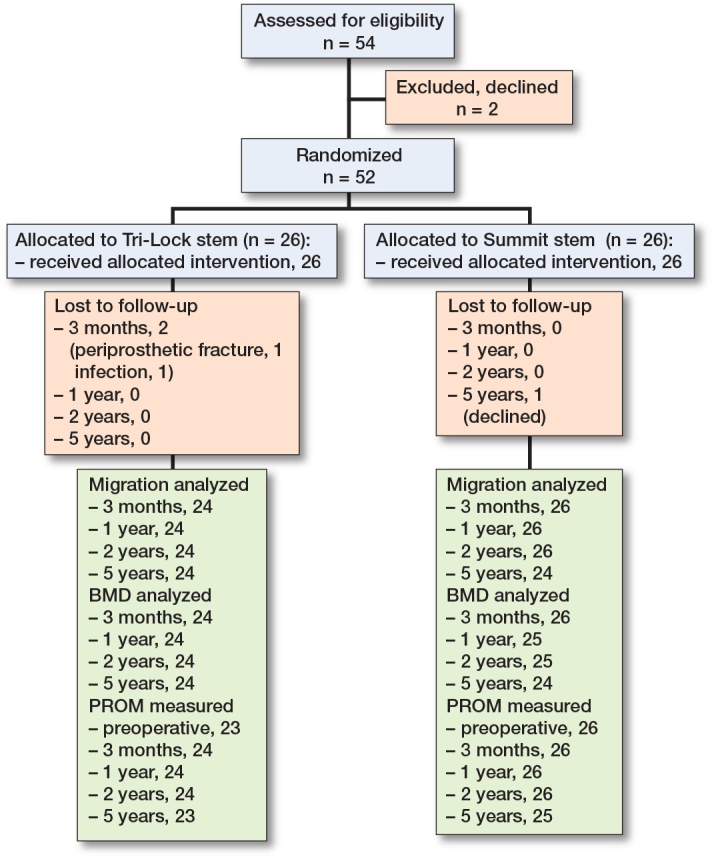
Consort flowchart.

Of 54 patients, 2 declined and 52 were randomized to Tri-Lock (n = 26) or Summit (n = 26) stems and received the allocated intervention. Between baseline and 3-month follow-up, 2 patients were excluded for clinical reasons. At 5-year follow-up, 1 additional patient withdrew and 1 RSA analysis was excluded for technical reasons. At 5-year follow-up, 24 patients in each group were analyzed for RSA and BMD changes (Figure 2 and Table 1).

**Table 1:** Baseline demographics

Item	Tri-Lock (n = 26)	Summit (n = 26)
Age, mean (SD)	59 (7)	62 (5)
Sex, male / female, n	21 / 5	37 / 15
T-score, mean (SD)	–0.8 (1.0)	–0.6 (1.1)
Normal/osteopenia/osteoporosis, n	15/10/1	19/6/1
BMI, mean (SD)	27 (4)	29 (4)
Oxford Hip Score, mean (SD)	26 (6)	24 (6)
EQ5D TTO, mean (SD)	0.69 (0.09)	0.64 (0.18)
Side, right / left, n	16 / 10	12 / 14
Head size, 32 / 36 mm, n **^a^**	26 / 0	24 / 2
Stem size, median (range)	5 (3–9)	5 (2–9)

a Cup size 50 mm was only available with a 32 mm femoral head.

### RSA

**Figure 3:**
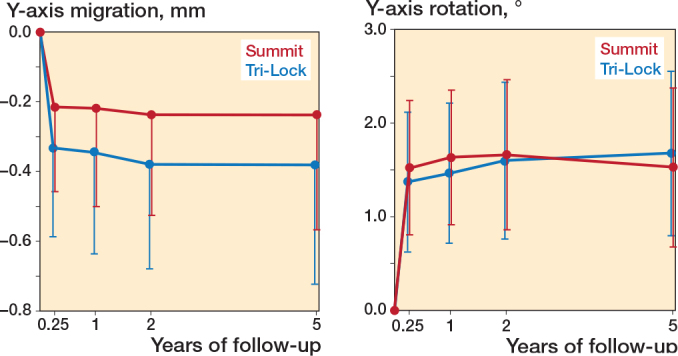
Subsidence (negative Y-translation) and retroversion (positive Y-rotation) of the Tri-Lock and Summit femoral stems.

At the 2-year follow-up, mixed model calculations showed no statistically significant difference between stem groups in subsidence (negative y-translation) (0.14 mm; CI –0.27 to 0.56) or retroversion (positive y-rotation) (0.06°; CI –1.10 to 1.22). Both stems showed subsidence and retroversion until 3-month follow-up and stabilized thereafter (Figure 3 and Table 2). At the 5-year follow-up, the Tri-Lock stems had subsidence of 0.38 mm (CI 0.04–0.72) and retroversion of 1.68° (CI 0.80–2.55). The Summit stems had subsidence of 0.24 mm (CI 0.09–0.57) and retroversion of 1.53° (CI 0.68–2.37). At the 5-year follow-up, stem z rotation was –0.66° (CI –0.99 to –0.33) for Tri-Lock and –0.32° (CI –0.63 to 0.00) for Summit stems. Continuous subsidence or retroversion after 2-year follow-up beyond the limit of agreement was detected in 3 Tri-Lock stems and 8 Summit stems.

**Table 2:** Stem migration presented as mean (CI)

Item	Tri-Lock n = 24	Summit n = 26	Difference, mean (CI)
x-translation (medial (+)/lateral (–) translation)			
3 months	0.23 (0.15 to 0.31)	0.15 (0.07 to 0.23)	–0.08 (–0.19 to 0.04)
1 year	0.22 (0.13 to 0.31)	0.15 (0.07 to 0.24)	–0.07 (–0.20 to 0.05)
2 years	0.24 (0.14 to 0.33)	0.16 (0.07 to 0.25)	–0.08 (–0.21 to 0.05)
5 years	0.26 (0.15 to 0.37)	0.15 (0.04 to 0.25)	–0.11 (–0.26 to 0.04)
y-translation (proximal (+)/distal (–) translation)			
3 months	–0.33 (–0.59 to –0.08)	–0.21 (–0.46 to 0.03)	0.12 (–0.23 to 0.47)
1 year	–0.34 (–0.64 to –0.05)	–0.22 (–0.50 to 0.06)	0.13 (–0.28 to 0.53)
2 years	–0.38 (–0.68 to –0.08)	–0.24 (–0.52 to 0.05)	0.14 (–0.27 to 0.56)
5 years	–0.38 (–0.72 to –0.04)	–0.24 (–0.57 to 0.09)	0.14 (–0.33 to 0.62)
z-translation (anterior (+)/posterior (–) translation)			
3 months	–0.11 (–0.22 to –0.01)	–0.04 (–0.14 to 0.07)	0.08 (–0.07 to 0.23)
1 year	–0.18 (–0.28 to –0.08)	–0.03 (–0.13 to 0.06)	0.15 (0.01 to 0.28)
2 years	–0.18 (–0.29 to –0.07)	–0.01 (–0.12 to 0.09)	0.17 (0.02 to 0.32)
5 years	–0.16 (–0.28 to –0.04)	–0.06 (–0.18 to 0.06)	0.11 (–0.06 to 0.27)
x-rotation (anterior (+)/posterior (–) tilt)			
3 months	0.01 (–0.18 to 0.20)	0.03 (–0.15 to 0.21)	0.02 (–0.24 to 0.28)
1 year	0.01 (–0.16 to 0.18)	–0.01 (–0.17 to 0.15)	–0.02 (–0.25 to 0.21)
2 years	0.08 (–0.12 to 0.28)	–0.04 (–0.23 to 0.15)	–0.12 (–0.40 to 0.16)
5 years	–0.05 (–0.31 to 0.21)	–0.20 (–0.46 to 0.05)	–0.15 (–0.52 to 0.21)
y-rotation (retroversion (+)/anteversion (–))			
3 months	1.37 (0.63 to 2.12)	1.52 (0.81 to 2.24)	0.15 (–0.88 to 1.18)
1 year	1.47 (0.72 to 2.21)	1.63 (0.92 to 2.35)	0.17 (–0.87 to 1.20)
2 years	1.60 (0.76 to 2.43)	1.66 (0.86 to 2.46)	0.06 (–1.10 to 1.22)
5 years	1.68 (0.80 to 2.55)	1.53 (0.68 to 2.37)	–0.15 (–1.37 to 1.07)
z-rotation (valgus (+)/varus (–))			
3 months	–0.53 (–0.80 to –0.25)	–0.29 (–0.55 to –0.02)	0.24 (–0.14 to 0.62)
1 year	–0.55 (–0.83 to –0.27)	–0.31 (–0.58 to –0.04)	0.24 (–0.15 to 0.63)
2 years	–0.55 (–0.85 to –0.25)	–0.31 (–0.60 to –0.03)	0.24 (–0.18 to 0.65)
5 years	–0.66 (–0.99 to –0.33)	–0.32 (–0.63 to 0.00)	0.35 (–0.11 to 0.80)
Total translation (TT) **^a^**			
3 months	1.75 (1.05 to 2.45)	1.91 (1.24 to 2.58)	0.16 (–0.81 to 1.13)
1 year	1.84 (1.09 to 2.58)	1.77 (1.05 to 2.49)	–0.07 (–1.10 to 0.96)
2 years	1.94 (1.14 to 2.75)	2.01 (1.24 to 2.79)	0.07 (–1.05 to 1.19)
5 years	2.16 (1.29 to 3.03)	1.82 (0.98 to 2.67)	–0.34 (–1.55 to 0.88)
Total rotation (TR) **^b^**			
3 months	0.60 (0.38 to 0.83)	0.53 (0.31 to 0.74)	–0.08 (–0.39 to 0.23)
1 year	0.63 (0.36 to 0.89)	0.53 (0.28 to 0.79)	–0.09 (–0.46 to 0.27)
2 years	0.67 (0.40 to 0.94)	0.57 (0.31 to 0.83)	–0.10 (–0.48 to 0.27)
5 years	0.72 (0.40 to 1.04)	0.57 (0.27 to 0.88)	–0.14 (–0.58 to 0.29)
Maximum total point motion (MTPM) **^c^**			
3 months	1.95 (1.31 to 2.59)	1.75 (1.13 to 2.37)	–0.20 (–1.09 to 0.69)
1 year	2.08 (1.37 to 2.79)	1.73 (1.05 to 2.41)	–0.35 (–1.33 to 0.63)
2 years	2.20 (1.45 to 2.94)	1.91 (1.20 to 2.62)	–0.29 (–1.31 to 0.74)
5 years	2.60 (1.75 to 3.46)	2.06 (1.23 to 2.88)	–0.55 (–1.74 to 0.64)

a Total translation denotes the combined translation resulting from the translation in each axis: Total translation = √(x-translation ^2^ + y-translation ^2^ + z translation ^2^)

b Total rotation denotes the combined translation resulting from the translation in each axis: Total rotation = √(x-rotation ^2^ + y-rotation ^2^ + z rotation ^2^)

c Maximum total point motion denotes the largest translation of a point of the implant resulting from the rotation and translation in combination.

2 Tri-Lock stems rotated more than –2° around the z-axes into varus before the 3-month follow-up and stabilized thereafter.

There were no statistically significant correlations between preoperative T-score and retroversion (β 0.08, CI –0.26 to 0.41) or subsidence (β 0.11, CI –0.23 to 0.45), or mean percentage 2-year change in BMD and retroversion (β –0.20, CI –0.55 to 0.14) or subsidence (β –0.18, CI –0.51 to 0.16).

### BMD

**Figure 4:**
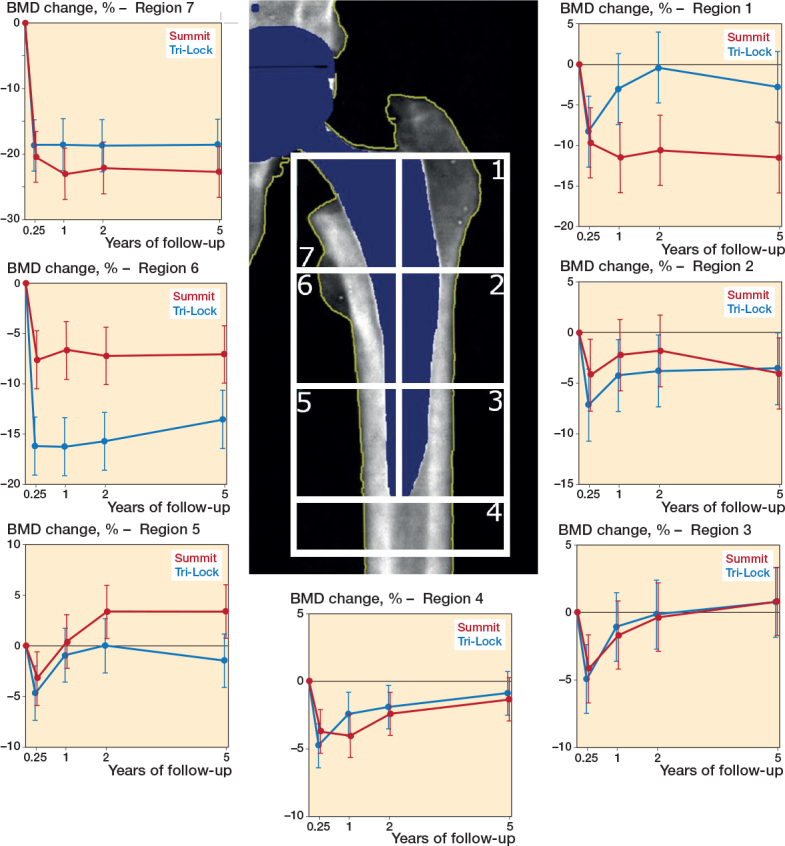
Change in BMD (%) of the 7 Gruen zones from the postoperative DXA-scan for the Tri- Lock (blue line) and Summit (red line) stems.

From baseline to 3-month follow-up, mixed model calculations showed a BMD loss in all 7 zones, but greatest in zone 7 (calcar) (Figure 4 and Table 3). From 3-month to 5-year follow-up, BMD improved or was unchanged in all zones for both groups.

**Table 3:** BMD percentage change from postoperative values in Gruen zones, given as mean (CI)

Item	Tri-Lock n = 24	Summit n = 26	Difference, mean (CI)
Zone 1			
3 months	–8.3 (–12.6 to –4.0)	–9.7 (–14.0 to –5.4)	–1.4 (–7.5 to 4.7)
1 year	–3.1 (–7.4 to 1.3)	–11.5 (–15.8 to –7.2)	–8.4 (–14.5 to –2.3)
2 years	–0.4 (–4.8 to 3.9)	–10.6 (–14.9 to –6.3)	–10.1 (–16.3 to –4.0)
5 years	–2.8 (–7.1 to 1.6)	–11.5 (–15.8 to –7.2)	–8.7 (–14.8 to –2.6)
Zone 2			
3 months	–7.1 (–10.7 to –3.6)	–4.2 (–7.7 to –0.7)	2.9 (–2.1 to 7.9)
1 year	–4.2 (–7.7 to –0.7)	–2.2 (–5.7 to 1.3)	2.0 (–3.0 to 6.9)
2 years	–3.8 (–7.3 to –0.3)	–1.8 (–5.3 to 1.7)	2.0 (–3.0 to 6.9)
5 years	–3.6 (–7.1 to 0.0)	–4.0 (–7.5 to –0.5)	–0.5 (–5.4 to 4.5)
Zone 3			
3 months	–4.9 (–7.5 to –2.4)	–4.2 (–6.7 to –1.7)	0.8 (–2.8 to 4.3)
1 year	–1.1 (–3.6 to 1.5)	–1.7 (–4.2 to 0.8)	–0.6 (–4.2 to 3.0)
2 years	–0.2 (–2.7 to 2.4)	–0.4 (–2.9 to 2.1)	–0.2 (–3.8 to 3.4)
5 years	0.7 (–1.8 to 3.3)	0.8 (–1.7 to 3.3)	0.1 (–3.5 to 3.7)
Zone 4			
3 months	–4.8 (–6.4 to –3.2)	–3.7 (–5.3 to –2.1)	1.1 (–1.2 to 3.3)
1 year	–2.5 (–4.1 to –0.8)	–4.0 (–5.7 to –2.4)	–1.6 (–3.9 to 0.7)
2 years	–1.9 (–3.5 to –0.3)	–2.4 (–4.0 to –0.8)	–0.5 (–2.8 to 1.8)
5 years	–0.9 (–2.5 to 0.7)	–1.4 (–3.0 to 0.2)	–0.5 (–2.7 to 1.8)
Zone 5			
3 months	–4.7 (–7.3 to –2.0)	–3.2 (–5.9 to –0.6)	1.4 (–2.3 to 5.2)
1 year	–0.9 (–3.6 to 1.7)	0.4 (–2.2 to 3.0)	1.3 (–2.4 to 5.1)
2 years	0.0 (–2.6 to 2.7)	3.3 (0.7 to 5.9)	3.3 (–0.4 to 7.0)
5 years	–1.5 (–4.1 to 1.2)	3.4 (0.8 to 6.0)	4.8 (1.1 to 8.6)
Zone 6			
3 months	–16.2 (–19.1 to –13.3)	–7.7 (–10.5 to –4.8)	8.5 (4.5 to 12.6)
1 year	–16.3 (–19.1 to –13.4)	–6.7 (–9.5 to –3.8)	9.6 (5.5 to 13.6)
2 years	–15.7 (–18.6 to –12.8)	–7.3 (–10.1 to –4.4)	8.5 (4.4 to 12.5)
5 years	–13.6 (–16.5 to –10.7)	–7.1 (–10.0 to –4.3)	6.5 (2.4 to 10.5)
Zone 7			
3 months	–18.7 (–22.6 to –14.7)	–20.4 (–24.3 to –16.5)	–1.7 (–7.3 to 3.8)
1 year	–18.5 (–22.5 to –14.6)	–23.0 (–26.9 to –19.1)	–4.5 (–10.0 to 1.1)
2 years	–18.7 (–22.6 to –14.8)	–22.1 (–26.0 to –18.2)	–3.4 (–8.9 to 2.2)
5 years	–18.6 (–22.5 to –14.6)	–22.7 (–26.6 to –18.8)	–4.2 (–9.7 to 1.4)

Throughout all follow-ups, the Summit stem preserved less bone in the metaphysis compared with the Tri-Lock stem, with a 5-year difference of –4.2% (CI –9.7 to 1.4) in zone 7 (calcar) and of –8.7% (CI –14.8 to –2.6) in zone 1 (greater trochanter).

Throughout all follow-ups, the Summit stem preserved more bone in the medial diaphysis compared with the Tri-Lock stem, with a 5-year difference of 4.8% (CI 1.1–8.6) in zone 5 and 6.5% (CI 2.4–10.5) in zone 6.

At 3-month follow-up, BMD loss in zones 1–6 ranged between –16.2% and –3.2% for both stem groups compared with baseline (see Table 3). In the calcar zone (zone 7), the mean BMD loss was –18.7% (CI –22.6 to –14.7) for Tri-Lock and –20.4% (CI –24.3 to –16.5) for Summit. In zone 6, the reduction was significantly larger for Tri-Lock –16.2% (CI –19.1 to –13.3) than for Summit –7.7% (–10.5 to –4.8).

From 3-month to 5-year follow-up, BMD improved or was unchanged in all zones for both groups. From baseline to 5-year follow-up, there was no statistically significant difference in BMD in zones 2, 3, 4, and 7.

### PROMs

For Oxford hip score, the Tri-Lock group improved from median 25 points (range 16–38) at baseline to 47 (range 30–48) points at 5-year follow-up, and the Summit group improved from 22 points (range 10–32) to 45 points (range 17–48) (P = 0.4). EQ5D and pain scores improved in both stem groups from baseline to 5-year follow-up (see Supplementary Figures 2 and 3).

### Implant-related complications

None of the femoral stems were revised due to aseptic loosening. 1 patient in the Tri-Lock group had a hip dislocation at 3-month follow-up, which was reduced closed and the patient remained in the cohort for all data analyses. 1 patient in the Tri-Lock group had a periprosthetic fracture, which was discovered on radiographs at 3-month follow-up and treated conservatively. The patient was excluded from all data analyses. 1 patient in the Tri-Lock group had a deep prosthetic joint infection, which was treated with one-stage revision and the patient was excluded from all data analyses.

## Discussion

This study aimed to compare fixation and periprosthetic BMD loss of the new Tri-Lock stem with the classic Summit stem. We showed no clinically important differences in stem migration between Tri-Lock and Summit stems at 2- and 5-year follow-up. BMD was best preserved for Tri-Lock stems in zone 1, and for Summit in zones 5 and 6. There was no difference in PROMs.

### Migration

This is the first RSA study to evaluate migration of the Tri-Lock and Summit femoral stems. Short stems, like the Tri-Lock stem, are designed with more or less of a banana shape and intended to preserve the bone of the femoral neck and trochanter. Early subsidence (2-year) for a similar titanium porous coated short stem (Fitmore) has been reported to be 0.39 mm and retroversion to be 1.09° [25]. These findings concur with our findings of 2-year subsidence and retroversion of mean 0.38 mm and 1.60° for the Tri-Lock stem.

At 5-year follow-up, the Collared Collum Femoris Preserving stem has been reported with a subsidence of 0.27 mm [26] and the collarless short neck-sparring titanium Metha stem with a taper-wedge design has shown 5-year subsidence of 0.71 mm and retroversion of 3.4° [27]. This is more than our findings of 0.38 mm subsidence and 1.68° retroversion a 5-year follow-up. In addition, Liu et al. have measured 5-year subsidence for both Tri-Lock (1.89 mm) and Summit (1.99 mm) using Einzel-Bild-Roentgen-Analyze [28]. This is more than we found for both the Tri-Lock (0.38 mm) and the Summit (0.24 mm) stems. The reason for the relatively high subsidence measured by Liu et al. could be that they included patients with more variation in the diagnosis leading to THA. The low subsidence for both stems found in this study is in concordance with the ODEP ratings of 13A (Tri-Lock) and 15A (Summit), indicating good long-term survival of both stems.

Cementless femoral stems have been shown to subside more in women with osteopenia and osteoporosis determined by a preoperative T-score below –1 [6]. The present study evaluated men and women with a preoperative T-score in the range of –2.8 to 1.3 and age range 46–70 years but did not find an association between stem subsidence and preoperative BMD or postoperative BMD loss in either of the stem groups. This is in agreement with the findings of Dyreborg et al. who studied the Echo Bi-Metric Full Proximal Profile cementless stem for a patient group with age range of 49–74 years and T-score range of –2.3 to 3.5 [29]. Thus, migration of cementless femoral stems with metaphyseal fixation seems to be unaffected by preoperative BMD, when this is in the normal-to-osteopenia category.

### BMD

There is evidence of reduced bone resorption of the metaphyseal area in the first 2 years after insertion of short femoral stems [30,31]. This is likely caused by more direct proximal loading in the greater trochanter area, leading to less bone stress shielding. The BMD measurements in the Tri-Lock group in our study confirm a low bone loss of –2.8% in the trochanter zone, but also show high medial diaphyseal bone resorption of –13.6% indicating a bone area of unloading. This is similar to findings in the Proxima short stem (–13%) but exceeds findings for medial proximal bone loss in the Metha stems (–3%). The reason for less medial-proximal bone resorption with the Metha stem may be found in the more pronounced curving of the stem leading to an increased load transfer to the medial-proximal zone [28,30,31].

The Summit stem is straight, round, and longer compared with the Tri-lock stem. During insertion of the Summit stem, bone is rasped and removed from the trochanter. BMD measurements show that at 3 months and continued throughout 5-year follow-up, stress shielding is high in the metaphysis with –11.5% in the trochanter area and –22.7% in the calcar area. Specifically, the BMD measurements of this study support distal fixation with the Summit stem with high loads on the bone in the medial diaphysis at the stem tip with an actual mean BMD improvement of 3.4% at 5-year follow-up.

### PROMs

The 5-year median EQ5D of 1 exceeded the patient- acceptable symptom state of 0.91 for both groups [32]. Likewise, the median OHS of 47 points (Tri-Lock) and 45 points (Summit) was better than the mean value for the reference population of 39.8 points [33]. Both scores were subject to ceiling effect. Pain scores were excellent in both groups and subject to floor effect.

### Strengths

The study strengths are the randomized patient-blinded design, the use of precise state-of-the-art measurement methods, and the very high patient compliance for follow-up. Furthermore, the external validity is good, as cementless implants are specifically intended for younger patients (in this study 46–70 years) without osteoporosis.

### Limitations

Because both stem types were made from a titanium alloy, the mechanical bone stimulus from the metal elasticity of the implants should be similar. Thus, the geometry of the implants has likely had the highest effect on the postoperative bone modulation [34]. However, the results for migration and BMD changes cannot be directly extrapolated to femoral stems of other metal alloys.

The sex distribution in this study was skewed towards male participants, which may have influenced the outcomes related to implant migration and changes in periprosthetic BMD. While sex-specific effects on these parameters remain underexplored, several studies have reported an increased risk of revision due to aseptic loosening in men compared with women. Conversely, estrogen deficiency is a well-established factor contributing to decreased BMD in patients with osteoporosis, and may therefore have a greater impact in female patients [35].

### Conclusion

The Tri-Lock and Summit stems showed similar migration until mid-term. Initially, peri-prosthetic bone mineral density was lost for both stem types. In the following years, the taper-wedge stem design regained metaphyseal BMD in the trochanteric area, while the long and round-tapered stem design regained or even increased diaphyseal BMD. In terms of stem fixation, bone resorption, and PROMs both stems performed well at mid-term.

### Supplementary data

Supplementary Tables 1–2 and Supplementary Figures 1–3 are available as Supplementary data on the article page, doi: 10.2340/17453674.2025.43907

## Supplementary Material



## Notes

PBJ: formal analysis, visualization, writing – original draft, writing – review & editing. MH: conceptualization, funding acquisition, investigation, validation, writing – review & editing. DK: validation, formal analysis, writing – review & editing. TBH: conceptualization, funding acquisition, supervision, writing – review & editing. MS: conceptualization, methodology, supervision, visualization, writing – original draft, writing – review & editing.

Handling co-editors: Keijo Mäkelä and Philippe Wagner

*Acta* thanks Johan Kärrholm and Sarunas Tarasevicius for help with peer review of this manuscript.
