# Supplementary material for: Similar femoral stem fixation but less metaphyseal loss of bone mineral density with a taper-wedge design and diaphyseal bone preservation with a long and round-tapered design: a 5-year randomized RSA and DXA study of 50 patients
Source: Acta Orthop. 2025 Sep 2;96:656–63. doi: 10.2340/17453674.2025.43907 (PMC12404101; doi:10.2340/17453674.2025.43907)
Supplement: Supplementary file 1 [file ActaO-96-43907-s1.pdf]

Supplementary data

Supplementary Table 1. Precision of radiostereometric analysis

Migration (n = 48)	Difference	Range	SD	Limit of agreement (+/–)
X-translation	0.01	–0.12 to 0.15	0.05	0.10
Y-translation	0.00	–0.16 to 0.31	0.09	0.18
Z-translation	0.00	–0.28 to 0.33	0.12	0.24
X-rotation	0.03	–0.85 to 0.77	0.26	0.51
Y-rotation	0.04	–1.16 to 0.92	0.40	0.78
Z-rotation	–0.02	–0.27 to 0.18	0.09	0.18
Total translation [a]	–0.02	–0.35 to 0.15	0.09	0.18
Total rotation [b]	0.01	–0.81 to 0.86	0.32	0.63
Maximum total point motion [c]	–0.03	–0.82 to 0.47	0.24	0.47

The precision of stem migration measurements was measured as the mean difference (SD) between the 2 recordings and reported with a min and max for all migration parameters [20].

[a] Total translation denotes the combined translation resulting from the translation in each axis:

Total translation = $\sqrt{x\text{-}translation^2 + y\text{-}translation^2 + z\text{-}translation^2}$

[b] Total rotation denotes the combined translation resulting from the translation in each axis:

Total rotation = $\sqrt{x\text{-}rotation^2 + y\text{-}rotation^2 + z\text{-}rotation^2}$

[c] Maximum total point motion denotes the largest translation of a point of the implant resulting from the rotation and translation in combination.

Supplementary Table 2. Precision of dual energy x-ray absorptiometry

Zone	Mean BMD (g/cm^2)	SD	Range	%CV
1	0.92	0.02	0.45 to 1.34	2.20
2	2.06	0.03	1.57 to 2.86	1.56
3	2.31	0.02	1.66 to 3.06	1.05
4	2.16	0.02	1.48 to 2.71	0.75
5	2.28	0.03	1.74 to 2.74	1.31
6	1.71	0.03	0.97 to 2.36	1.82
7	1.28	0.05	0.91 to 1.74	3.79

Precision of periprosthetic BMD measurements was calculated using the root-mean-square approach and presented as % coefficient of variation (CV) [35].

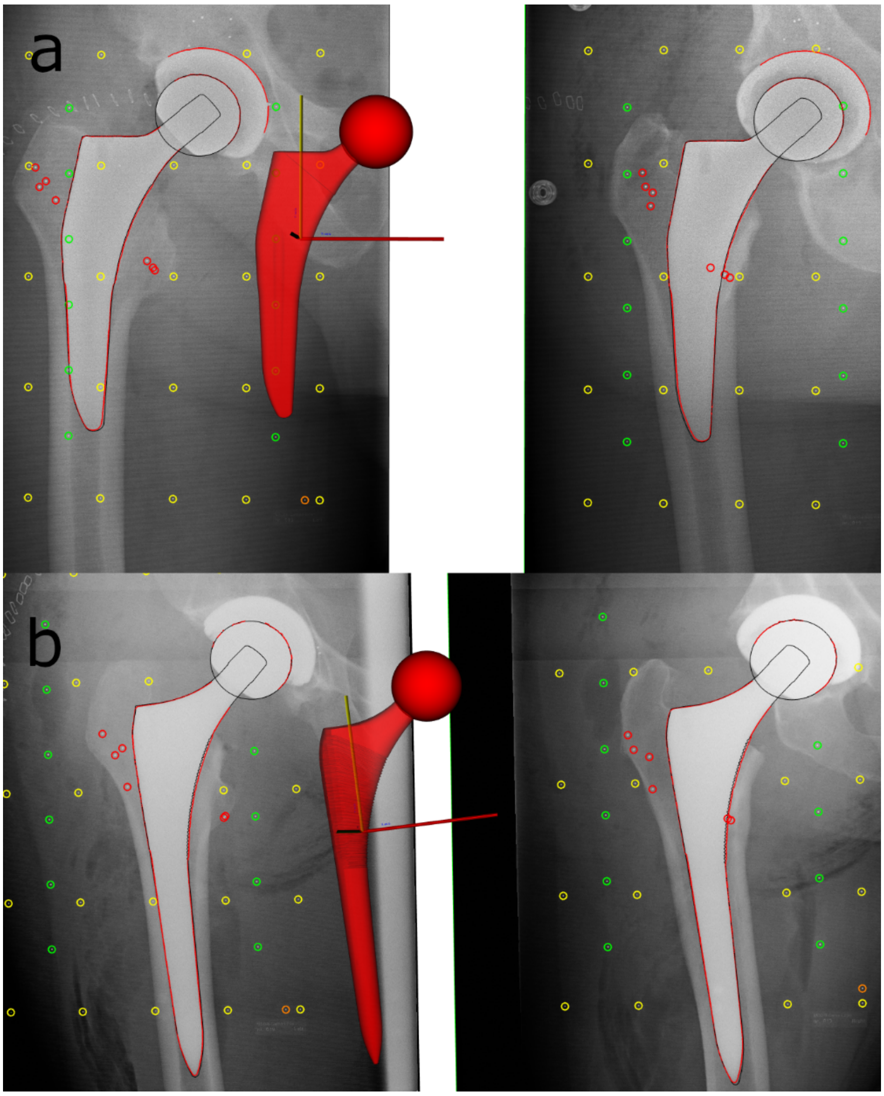

Supplementary Figure 1. Coordinate systems of the Tri-Lock (a) and Summit (b) stems showing the x- (red), y- (yellow) and z-axis (green) of the model in the RSA analysis.

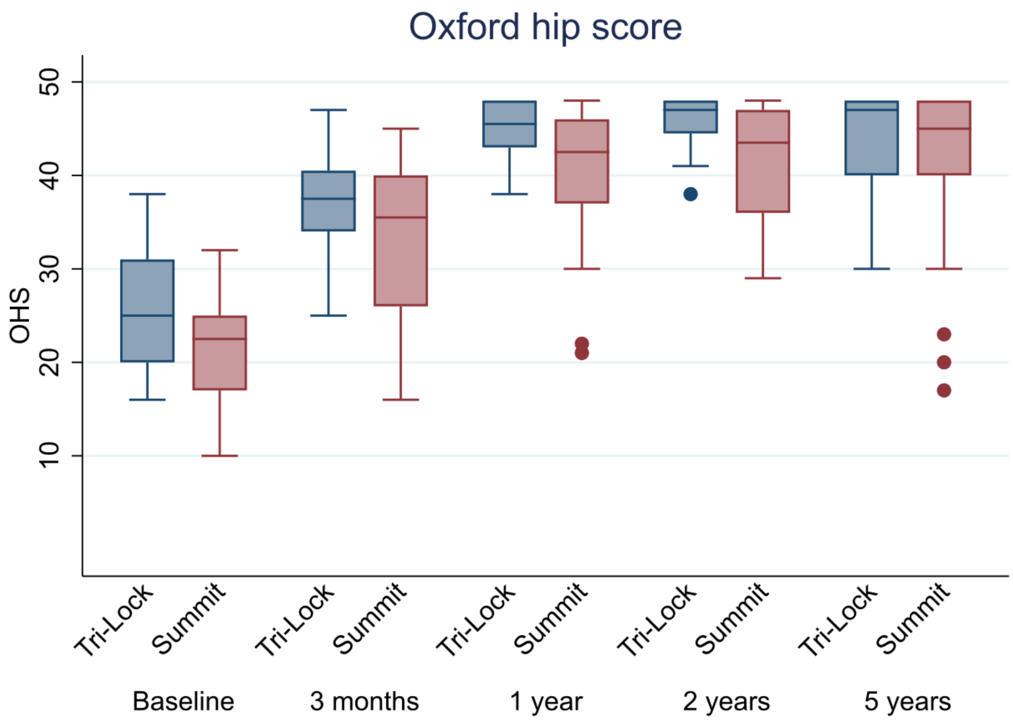

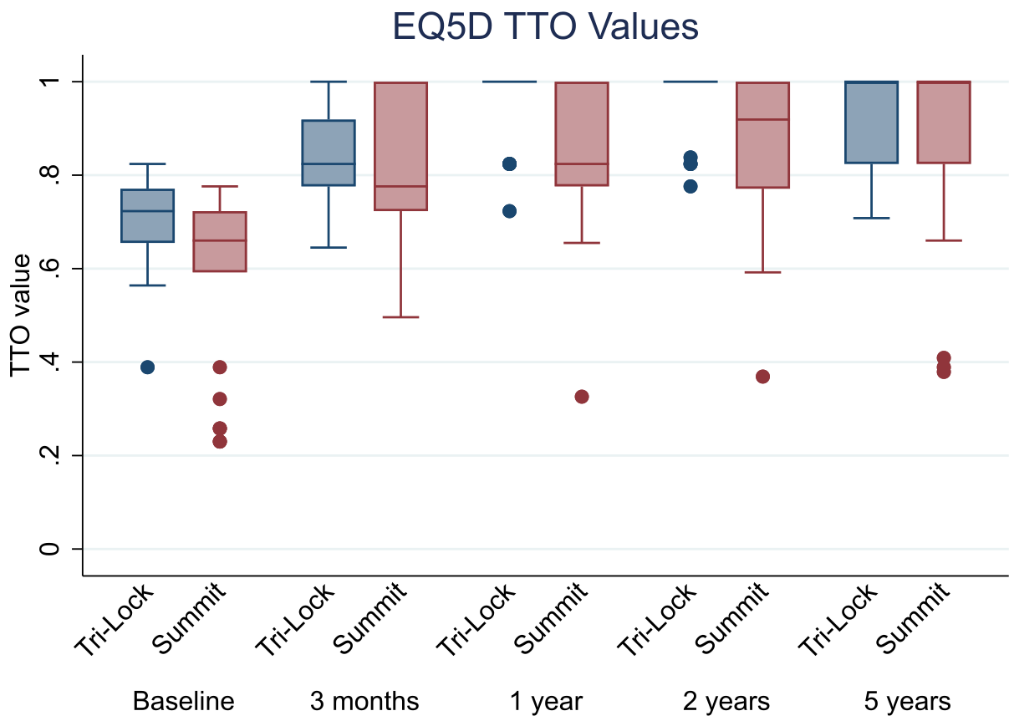

Supplementary Figure 2. Oxford Hip Score and EuroQual5D.

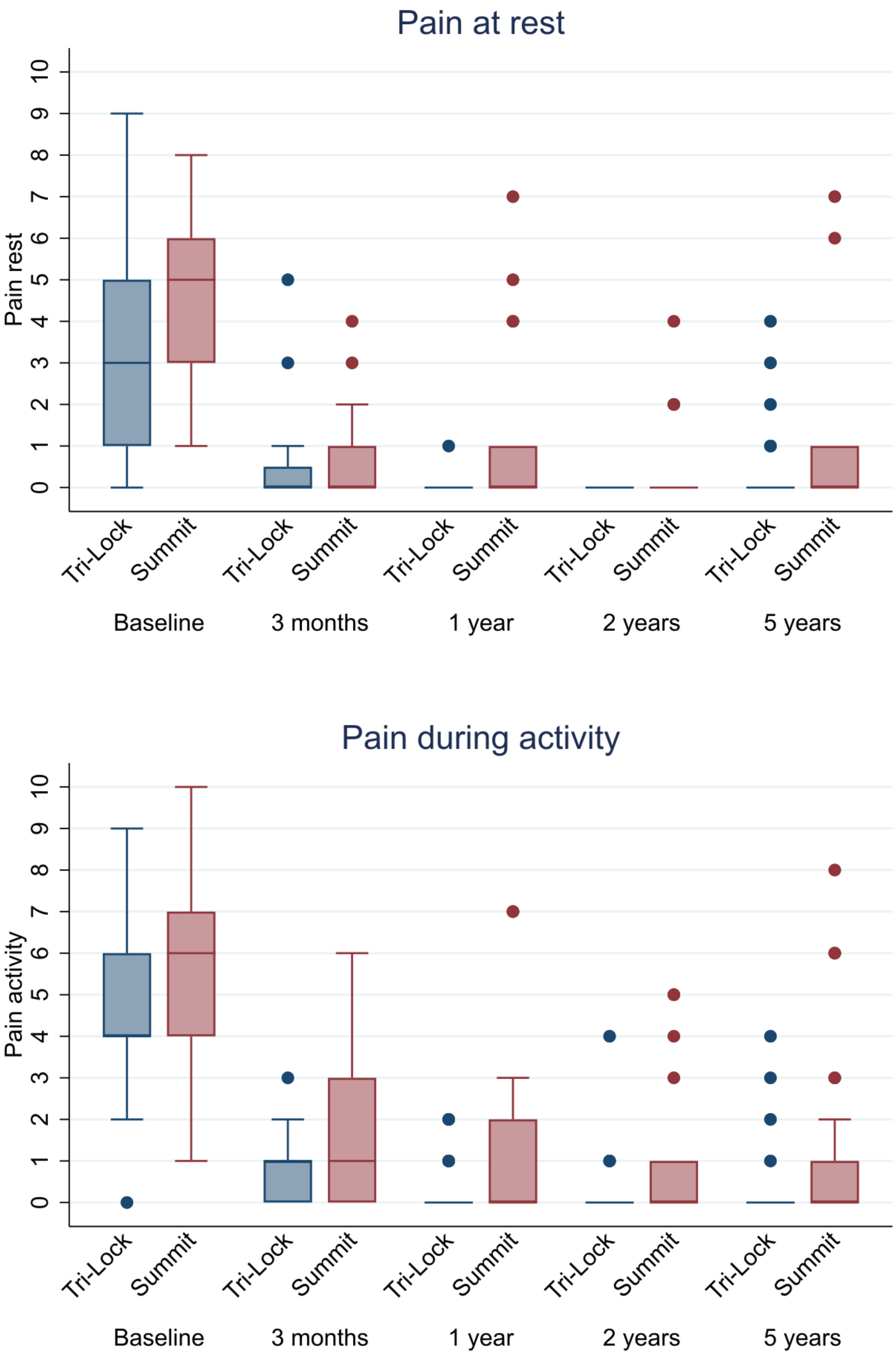

Supplementary Figure 3. Pain at rest and during activity.
